# The Athenian Epidemic of Meningitis of 1911

**Published:** 1912-03

**Authors:** 


### The Athenian Epidemic of Meningitis of 1911.

CONSTANTINUS M. MERCURIUS, M.D., in the *Medical Progress* (*Hiatrike Prohodos*), of August, 1911. Specially translated for the BUFFALO MEDICAL JOURNAL from the original Greek.

In the current epidemic of cerebro-spinal meningitis, beginning suddenly in the month of January, already disappearing, about 20 patients were entirely under my care or were seen in consultation. Thirty other indubitable cases occurred among the approximately 18,000 inhabitants of the two neighboring Athenian districts, Cropia and Thoricii. The present article deals only with the 20 personally seen. (Note: We have taken the liberty, occasionally to condense and to make a free translation.)

Of the 20 cases, 13 were females, only 7 males, 2 died of each sex—28 per cent. males, 16 per cent. females. According to age, the incidence and mortality were as follows:

| | | |
|---|---|---|
| 3 months—1 year | 3 cases | 1 death |
| 1—3 years | 4 cases | 1 death |
| 3—10 years | 3 cases | 1 death |
| 11—19 years | 9 cases | 0 deaths |
| 35 years | 1 case | 1 death |

### CAUSE AND ORIGIN.

Local causes. The exceptionally severe preceding winter with frequent and persistent rains is noteworthy. Nor can the opinion as to the influence of cold and dampness on recrudescence and communicability be ignored.

General causes. 1. In all of the 20 cases, the houses were near the ground. 2. In two-thirds of the cases, the houses were damp, badly ventilated, dark, and had narrow and filthy halls. 3. The first three conditions were noted in the remaining third.

Transmission and Prophylaxis. Quarantine was inforced from beginning to end of all cases. Immediate contagion did not occur. Two critical cases in the same house broke out at the same time. Thymol mouth washes, borax and menthol ointment for the nostrils, especially for fissures of the mucous membrane or skin, sacks of naphthalene suspended from the neck, were employed.

Peculiarities. In a girl of three, the disease ran like a thunderbolt to a fatal issue. In the three other fatal cases, there were already anaemia and cachexia from chronic malaria and the intercurrent meningitis was severe. However, death was delayed by the specific serum and occurred in coma, without fever, after the maintenance of a normal pulse for a considerable time.

Symptomatology. The inception was almost always violent, with the classic tripod of symptoms and for the most part, without antecedent nasal catarrh. The fever was always high, exceeding 39 (102.2 F.), sustained in the beginning, then intermitting and rising again in the beginning, then intermitting and rising again in the evening after a lapse of 8—10 days. The pulse was from 80 to 130, in one case maintaining a rate of 150 or more persistently till convalescence. Slow pulse did not occur once from compression. Spasm of the masseters was observed in only two cases.

The signs of Kernig, Babrosky and Brudzinski were present in all cases. Labial herpes was found in 80 per cent. of cases. In an 18-year old girl, it was accompanied for the first few days, before any injection of lymph, by a severe albuminuria—10—12 grams a day—with epithelial casts, corresponding to the exacerbations of the fever and disappearing almost exactly at the time of defervescence. To the renal condition, I ascribed the marked rapidity of the pulse, the subacute pulmonary oedema and the protracted digestive disturbance which complicated the case.

Treatment. 1. As a routine, leeches were applied over the mastoids, at the beginning of the disease. Headache, delirium and digestive disturbances were relieved and the pulse became regular. Most drugs given by mouth, including bitter cathartics, and enemata of sodium sulphate, were usually rejected. Spinal blisters were not used. Intra-spinal injections of electrargol seemed to be of some value. Crede ointment did not hit the mark, though used perseveringly in the protracted cases. I regard this remedy as something to be dropped from the category of therapeutic measures against meningitis. The moderate, rational use of lukewarm baths, carried out under the physician's supervision, greatly relieved the suffering. The application of ice and salt to the head, by a bag and along the spine by a rubber cylinder, acted well.

2. Three forms of anti-meningitic sera were employed. That of Dopter-Pasteur, succeeded in recent cases in accomplishing a cure in the shortest time. Injected intra-spinally, in a three-year old boy, within 36 hours from the inception of the disease, in 10 c.c. dose, it completely removed the symptoms in a period of scarcely four hours. Six to eight doses, during a week, brought the cases to recovery. In the severest cases, it reduced the temperature to normal but did not save the patients.

The Merck serum also demonstrated its efficacy, quickly controlling the symptoms in a three year old child, in 10 c.c. dose, on the fourth day and producing a cure in 12 hours. The best result was obtained by the preliminary withdrawal of a considerable quantity of endo-cranial liquid, about double the bulk of the serum to be injected. For instance, in an 18-year old youth, 15 c.c. were withdrawn, 10 c.c. of serum injected and the advantage was quickly shown. But, on account of the accompanying nephritis and continued rapid pulse, the three forms of sera were tried, to the amount of 80 c.c. altogether. The case finally recovered after a month and a half. The specific treatment merely mitigated the symptoms and various adjuvant measures were of little effect so that we lost hope. Finally, Merck's iodipine cut short the apparently endless course of the disease. We began the use of this drug empirically in the minimum dose of 5 c.c. The case defervesced and other symptoms disappeared in 48 hours. Whatever headaches and minor rises of temperature reappeared, were easily controlled by tablets of iodipine and no further medication was found necessary There was no preconceived prejudice in favor of this drug. It proved absolutely harmless, not causing renal complications, so that there is nothing to contra-indicate its wider use.

Among the cases of recovery, there remained no limited paralyses, local atrophies nor alterations of the special senses.

(Note. It may be of interest to note that Greek is by no means a dead language. While the pronunciation has changed materially and there have been various minor changes, the written language is essentially the same as it was 2,500 years ago. Imagine a German, who had studied Chaucerian English as a matter of grammar and translation in school, who had become extremely rusty on this slight knowledge of English and had then attacked a modern English medical article. The latter would be almost unintelligible, yet there is a difference in time of only about 500 years. Indeed the English language is scarcely 700 years old altogether.)

